# Do interventions containing risk messages increase risk appraisal and the subsequent vaccination intentions and uptake? – A systematic review and meta‐analysis

**DOI:** 10.1111/bjhp.12340

**Published:** 2018-09-17

**Authors:** Joanne E. Parsons, Katie V. Newby, David P. French

**Affiliations:** ^1^ Coventry University UK; ^2^ University of Manchester UK

**Keywords:** randomized controlled trial, risk appraisal, uptake, vaccination

## Abstract

**Purpose:**

There is good evidence that for many behaviours, increasing risk appraisal can lead to a change in behaviour, heightened when efficacy appraisals are also increased. The present systematic review addressed whether interventions presenting a risk message increase risk appraisal and an increase in vaccination intentions and uptake.

**Method:**

A systematic search identified randomized controlled trials of interventions presenting a risk message and measuring risk appraisal and intentions and uptake post‐intervention. Random‐effects meta‐analyses investigated the size of the effect that interventions had on vaccination risk appraisal and on vaccination behaviour or intention to vaccinate, and the size of the relationship between vaccination risk appraisal and vaccination intentions and uptake.

**Results:**

Eighteen studies were included and 16 meta‐analysed. Interventions overall had small significant effects on risk appraisal (*d* = 0.161, *p* = .047) and perceptions of susceptibility (*d* = 0.195, *p* = .025), but no effect on perceptions of severity (*d* = −0.036, *p* = .828). Interventions showed no effect on intention to vaccinate (*d* = 0.138, *p* = .195) and no effect on vaccination behaviour (*d* = 0.043, *p* = .826). Interventions typically did not include many behaviour change techniques (BCTs), with the most common BCT unique to intervention conditions being ‘Information about Health Consequences’. Few of the included studies attempted to, or successfully increased, efficacy appraisals.

**Conclusions:**

Overall, there is a lack of good‐quality primary studies, and existing interventions are suboptimal. The inclusion of additional BCTs, including those to target efficacy appraisals, could increase intervention effectiveness. The protocol (CRD42015029365) is available from http://www.crd.york.ac.uk/PROSPERO/.

**Statement of contribution**

***What is already known on this subject?***

Previous research indicates that an increase in risk appraisal is associated with increased uptake in health‐related behaviours.

Research suggests that interventions increasing risk appraisal have a greater effect on intention when elements of efficacy appraisals are simultaneously increased.

***What does this study add?***

- This is the first systematic review to examine the effect of interventions on risk appraisal and vaccination uptake using only experimental studies.
- Limitations of the interventions themselves, and those caused by study methods and reporting, mean that the potential value of this type of review is lost. Instead, its value is in shining a light on the paucity of experimental studies in this area, and the quality of methods and reporting used.
- Future experimental studies should examine interventions that focus exclusively on increasing risk and efficacy appraisal compared to controls, use conditional measures of risk, and improve reporting to enable both more accurate coding of intervention content and more accurate assessments of study bias.

## Background

Many infectious diseases are preventable through vaccination. Vaccinations are responsible for preventing two to three million deaths per year globally (WHO, 2016). The efficacy of vaccination can be demonstrated by the eradication of smallpox worldwide over the last 40 years (Miller & Sentz, 2006). Furthermore, in the United Kingdom, vaccination has led to a 99% reduction in meningitis C cases in those under 20 years old since its introduction in 1999 (NHS Choices, 2016).

Despite benefits to health at the individual and societal levels, uptake of vaccination does not reach targets set by the World Health Organization (WHO). It is estimated that 18.7 million children worldwide do not receive the recommended, routine vaccinations against preventable diseases (WHO, 2016). In developed countries, programmes routinely include vaccination against major childhood illnesses and vaccination against seasonal illnesses for groups at higher risk. In the United Kingdom, although free routine vaccinations are available for groups at higher risk, national uptake targets of these vaccinations are not met (WHO, 2016). Uptake levels of some vaccinations remain poor; for example, only 45.1% of adults under 65 years in a clinical risk group (i.e., those that are considered to be more at risk of the illness being vaccinated against, excluding pregnancy) in the United Kingdom received the flu vaccination in the 2015–2016 season (www.gov.uk).

Individual factors contribute to vaccination decisions, notably risk appraisal, defined as individuals’ beliefs about personal susceptibility associated with a disease and the severity of that disease (Wright, 2010). In a recent systematic review, vaccination uptake was lower amongst people who believed that they were unlikely to contract the disease or those who believed that the disease was not severe (Bish, Yardley, Nicoll, & Michie, 2011). Vaccination uptake was also lower when individuals believed that the vaccine was ineffective.

There is now good systematic review evidence that increasing risk appraisal can have a small effect on increasing behaviour and that interventions increasing risk appraisal have a greater effect on intention when elements of efficacy appraisals (comprised of self‐efficacy and response efficacy) are simultaneously increased (Peters, Ruiter, & Kok, 2013; Sheeran, Harris, & Epton, 2014; Tannenbaum *et al*., 2015). In line with this, one way of increasing vaccination uptake would therefore be to increase individuals’ beliefs about the risk of infectious diseases, and the efficacy of vaccinations in reducing that risk.

Existing meta‐analyses of experimental studies examining the effect of changing risk appraisals on behaviour have typically examined effects across a number of health‐related behaviours (Sheeran *et al*., 2014; Tannenbaum *et al*., 2015). This approach increases the number of studies analysed and thereby increases the strength of confidence in the effect size reported. By contrast, studies examining only one behaviour are considered more informative for developing future interventions, as estimates of effect can be reliably attributable to that one behaviour (Wright, 2010). In line with this, the systematic review of Brewer *et al*. (2007) included only studies of vaccination. This review however included cross‐sectional and prospective studies, which are not as informative for intervention design as experimental designs, as correlation alone does not allow causal relationships to necessarily be inferred (Weinstein, Rothman, & Nicolich, 1998).

A further meta‐analysis by Sheeran *et al*. (2014) examined the effect of heightening risk appraisal on intentions and behaviour. The overall effect (intention; *d* = 0.31, behaviour; *d* = 0.23), and the effect by behaviour type (including for vaccination: intention: *d* = 0.38; behaviour: *d* = 0.33), was reported. This meta‐analysis however only included randomized controlled trials that were successful in changing risk appraisals; if there was no change in risk appraisals, then they were not included in the review. This decision was taken by the authors because they specifically wanted to examine the relationship between risk and behaviour, necessitating that only studies where the manipulation of risk was successful be included. This however means that the success of existing interventions in changing risk appraisals cannot be inferred from the findings.

The primary aim of the present systematic review was to examine interventions reported in the literature to see whether those that include risk messages have been successful in influencing risk appraisals and the subsequent intentions and uptake of vaccination. To further add to the body of evidence about the relationship between risk appraisal and vaccination uptake, the secondary aims of the current systematic review were to examine the size of the relationship between these variables and to examine whether changes to risk appraisal are enhanced by experimentally induced increases in efficacy appraisal. It is the first systematic review to examine whether risk messages influence risk appraisal and vaccination using only experimental studies. This will enable firmer conclusions to be drawn about success of existing intervention strategies in changing risk and subsequent vaccination behaviour. The present systematic review also aimed to establish which BCTs were present in interventions used to increase risk appraisal and vaccination intention and uptake in the included studies, and how these were associated with changes in risk appraisal and vaccination intention and uptake.

## Method

This systematic review was conducted in accordance with the protocol (CRD42015029365) published on the International Prospective Register of Systematic Reviews (PROSPERO, http://www.crd.york.ac.uk/PROSPERO/).

### Inclusion and exclusion criteria

Studies were required to be randomized controlled trials, with random assignment of participants to experimental conditions. At least one control condition was required; this could have been either no intervention or usual practice. No date restrictions or limitations on country of study were set, but studies had to have been published in the English language.

Studies were included in the systematic review if they described an intervention aiming to increase vaccination intention or uptake that included a risk message. Whether an intervention had targeted an increase in risk appraisal was determined by whether this construct (namely susceptibility and/or severity) was measured and reported post‐intervention. Studies were also required to have measured vaccination uptake, or intention to have a vaccination, at least once following the intervention, where vaccination was the participant's own decision, not a decision made on the behalf of someone else, for example, a child.

To be included, studies had to include all of the necessary statistical information to calculate an effect size for changes in risk appraisal and vaccination intention or behaviour following the intervention. Where this information was not available, attempts were made to contact authors for appropriate data. If this was unsuccessful, then the study was included in the systematic review, but excluded from the meta‐analysis. Studies included in the systematic review were required to provide a description of the intervention (which could be any type or length of exposure). Where there was no description, or the information provided was not sufficiently reported, then attempts were made to contact authors for this information. In cases where no further intervention information was available, the available information was coded. Where no information on the intervention was available, the study was excluded from the systematic review.

### Search strategy

Peer‐reviewed publications were searched using CINAHL, MEDLINE, PsycINFO, Scopus (including Science Direct), and Web of Science. Reference sections of included papers were examined to identify any relevant studies that were not identified by the initial search. Forward citation searches were conducted on included articles and major systematic reviews in this area (namely Brewer *et al*., 2007; Sheeran *et al*., 2014; Tannenbaum *et al*., 2015). Last searches were completed in September 2017. Full search terms can be found in Appendix S1.

To identify unpublished studies the Ethos database was used to search for relevant PhD theses using combinations of the same search terms. In addition, the authors of included studies were contacted to identify any other unpublished, relevant studies (contact details for authors of eight studies were available, and of those, three responses were received). Furthermore, requests were distributed electronically via affiliated groups (namely European Association of Social Psychology, European Health Psychology Society, Midlands Health Psychology Network, Social, Personality and Health Network, and Society for Personality and Social Psychology) asking members if they were aware of any unpublished papers meeting the inclusion criteria.

### Screening

**Figure 1:**
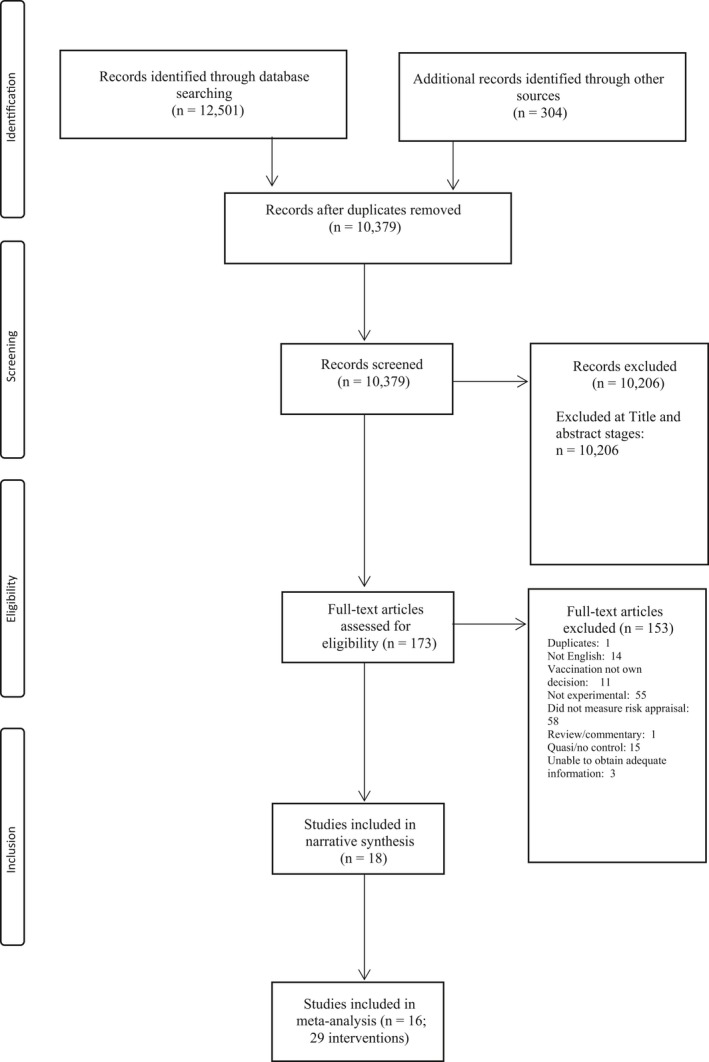
Flow chart of included studies.

Titles and abstracts of papers identified from database searches were initially screened by the lead author. A second stage of screening was undertaken using the full text of all studies that had not yet been excluded. This led to a sample of studies which met all inclusion criteria and which would provisionally be included in the meta‐analysis (see Figure 1). All studies considered eligible for inclusion, including any studies where inclusion was not clear, or where queries arose, were examined by the second author. A small number of minor discrepancies were resolved by discussion and a consensus reached on included studies.

### Extraction and coding

Information required for the calculation effect sizes was extracted. In all studies except one (Prati, Pietrantoni, & Zani, 2012), outcome data for susceptibility or severity or both were reported separately. In the study by Prati *et al*. (2012), a combined risk outcome measure was reported. All of this information was extracted. In addition, information was extracted for vaccination behaviour and intention to vaccinate. In studies that used multiple follow‐up measures, the first measure of risk and intention following intervention and the last measure of behaviour reported were used.

A number of study and sample characteristics were coded including the following: the illness type under examination, whether participants were pregnant, and the age group of participants. Whether interventions had successfully increased efficacy appraisals was also extracted. Please note that whilst it was originally planned that analysis would differentiate between increases in self‐efficacy and response efficacy, this was not possible. Of the three studies that successfully manipulated efficacy appraisals, only two measured self‐efficacy, and the other measured response efficacy and self‐efficacy as a combined measure. For this reason, efficacy appraisals were analysed as a combined measure. Age group was categorized as follows: adolescent: 16–18; adults: 19–64; and older adults: 65+. In cases where the age groups of participants in any one study crossed these boundaries, the age group was deemed to fall into the category where the majority of the participants resided. The nature of questions used to measure risk was also extracted to identify whether conditional or unconditional questions were used. Conditional questions refer to the likelihood of the event occurring according to whether action is taken to prevent it. Unconditional questions on the other hand refer to the likelihood of the event occurring regardless of action and take into account any subjective factors that influence the individual (van der Velde, Hooykaas, & van Pligt, 1996). Unconditional questions have been described as being methodologically inferior because they allow for the behavioural intentions of participants to influence risk appraisals (Weinstein *et al*., 1998).

Coding of BCTs within interventions was completed using the 93‐item Behaviour Change Technique Taxonomy v1 (Michie *et al*., 2013). Full interventions were coded where available, with authors being contacted for full interventions when these were not present within the paper. When no further information was provided, descriptions within the papers were coded. BCTs within both experimental and control group interventions were coded. Any BCTs that were present in both of the conditions were excluded to ensure that only unique intervention content was isolated. BCT coding was completed independently by both the lead author (who has previous experience in coding behaviour change techniques [BCTs]) and the second author (who has more extensive BCT coding experience). Any disagreements were discussed and a consensus was reached where required.

In addition, the lead author coded the following: the dose of each BCT (dose was derived from information available within intervention descriptions and was calculated by counting the number of times the BCT was delivered, using either the same intervention strategy or a something different), practical applications (Bartholomew Eldredge, 2016) used to deliver each BCT, and the mode of intervention delivery (in line with the Mode of Delivery of Behaviour Change Interventions Taxonomy version 0; Carey *et al*., 2016). Categorized modes included the following: printed material (‘Delivery through information produced on paper; can be hand‐delivered or posted to the participant; materials can include diagrams, pictures and text’); Digital: computer/television (‘Delivery through a computing device or television set’); and human: face‐to‐face (‘Delivery through human contact in which the participant meets a person in real‐time, face‐to‐face’). See Table S1.

### Assessment of study risk of bias

A risk of bias assessment is designed to assess the validity of included studies, and to examine whether any bias exists (whereby the true effect of the intervention is overestimated or underestimated). The Cochrane Risk of Bias Tool was used to assess the risk of bias in the included studies, and to assess the quality of the randomized controlled trials (Higgins & Green, 2011). Risk of bias assessment was completed by the lead author and independently assessed by a second coder. Any disagreements in scoring were discussed, and a consensus was reached.

Publication bias (the tendency for studies reporting significant or positive findings to be published more commonly than those without statistical significant findings, leading to meta‐analyses missing some studies) was assessed using funnel plots and trim‐and‐fill analysis conducted in line with Duval and Tweedie (2000).

### Statistical methods

Meta‐analysis software Comprehensive Meta‐Analysis (CMA) version 3 was used to calculate standardized mean difference for each intervention using a random‐effects model. Where separate outcome measures for risk were provided (i.e., susceptibility and severity), these were entered separately into CMA and their mean used within effect size calculations. A pooled and weighted standardized mean difference was thus calculated for risk (susceptibility and severity combined), intention to vaccinate, and behaviour (having the vaccination). Effect size estimates were however also calculated separately for measures of susceptibility and severity where studies provided the necessary information. Where studies included multiple interventions containing different types of risk messages, all of these interventions were included separately and the sample size of the control group was reduced to control for multiple comparisons. The relationship between risk appraisal and vaccination intention was assessed using a pooled, within‐study Pearson correlation coefficient. It was originally planned that the relationship between risk appraisal and vaccination uptake and between risk appraisal and intention to vaccinate would be examined. There were however insufficient studies reporting the relationship between risk appraisal and behaviour for the effects to be pooled. For this reason, only the relationship between risk appraisal and intention to vaccinate is reported. The heterogeneity of the results was calculated using the *I*² statistic (Higgin, Thompson, Deeks, & Altman, 2003).

A number of pre‐specified meta‐regression analyses were conducted. Moderators were only tested when they contained a sufficient range of values; that is, they had to be present or absent in at least three studies. Between‐group heterogeneity was assessed using the *Q* statistic to determine which moderators accounted for significantly different effect size estimates. Meta‐regression analysis was conducted to establish whether effect sizes for risk differed as a function of whether efficacy appraisal was also increased and whether conditional or unconditional questions of risk were used. In addition, they were conducted to establish whether effect sizes for risk appraisal or vaccination intention or uptake differed as a function of the illness being vaccinated against, the age group of participants, and whether study participants were pregnant or not.

A further pre‐specified meta‐regression analysis was also conducted to explore whether there was a difference in the size of effect (risk, intention, and behaviour) as a function of BCTs most commonly coded within the included interventions: Information about Health Consequences, Information about Social and Environmental Consequences, or Credible Source.

Two further meta‐regression analyses were performed that were not pre‐specified in the review protocol. These established whether there was a difference in the size of effect when more than two BCTs were included in the intervention, and according to the mode of delivery employed.

Moderators were only tested when they were present or absent in at least three studies. Accordingly, meta‐regression was not conducted for the following moderators: credible source for the outcome variable risk and credible source and number of BCTs for the outcome variable intention to vaccinate. No moderators were run for the outcome variable behaviour. The limited number of studies measuring behaviour meant that there were always too few studies with the moderator either present or absent.

## Results

Of 10,379 potential studies initially identified (after duplicates were removed), 18 satisfied all inclusion criteria. A table listing all included studies and summary characteristics can be found in Table S2. The majority of studies had a high percentage of female participants, with six studies involving female participants only, in part attributable to the nature of some studies examining vaccination intention or uptake in pregnancy. Only three studies recruited only men. Nine of the 18 included studies reported the mean age of participants, or the age range of participants, as being under 26 years. Seventeen of the 18 included studies were conducted in community settings. Community settings included participant's own homes, health centres, and churches. The remaining study (Gerend & Shepherd, 2012) was conducted in a laboratory within a university. Four studies used conditional risk questions, whereas 14 used unconditional risk questions (an example of a conditional risk question used is ‘What is the likelihood that you will get the flu this year if you don't get a flu shot?’ (Prati *et al*., 2012)) (Table 1).

**Table 1:** Summary table of frequency of characteristics of included studies

Characteristic	Number of studies
Study country	
USA	11
Other (non‐US country)	7
Illness being vaccinated against	
Flu	8
HPV	6
Hepatitis B	2
Flu and pneumococcal	1
Tetanus	1
Participants pregnant or not	
Pregnant	5
Not pregnant	13
Measure of risk	
Composite	12
Single	6

### Results of main outcomes

On the whole, studies reported a statistically significant increase in risk appraisal following intervention. Of the 18 included studies, 13 did not measure or manipulate efficacy appraisals. Of the five that did attempt to manipulate efficacy appraisals, three showed a statistically significant increase. Thirteen of the included studies measured intention as the primary outcome variable, whilst five studies measured behaviour as the primary outcome variable. Thirteen studies reported a statistically significant increase in vaccination uptake or intention to vaccinate post‐intervention. Five reported no increase in intention or uptake as a result of the intervention.

#### Meta‐analysis

Sixteen studies, reporting on the effect of 29 interventions, were able to be included in the meta‐analysis (Bennett *et al*. (2015) and Dabbs and Leventhal (1966) contained insufficient statistical information to be included in the meta‐analysis). A full table of effect sizes can be found in Table S3 and raw data files can be found in Supporting Information Tables S7–S12.

Study interventions had a small but significant pooled effect on risk appraisal (*d* = 0.161, 95% CI .002 to .320, *n* = 7,914, *k* = 29, *p* = .047, *I*² = 76.855). By contrast, there was no significant pooled effect on intention (*d* = 0.138, 95% CI −0.071 to 0.346, *n* = 5,905, *k* = 19, *p* = .195, *I*² = 72.613) or on behaviour (*d* = 0.043, 95% CI −0.343 to 0.429, *n* = 2009, *k* = 9, *p* = .826, *I*² = 79.468). Interventions had a small significant pooled effect on susceptibility (*d* = 0.195, 95% CI 0.024 to 0.366, *n* = 6722, *k* = 27, *p* = .025) but no pooled effect on severity (*d* = −0.036, 95% CI −0.366 to 0.293, *n* = 5390, *k* = 15, *p* = .828). There was a small significant relationship (*r* = .114, 95% CI = 0.031 to 0.196, *n* = 1017, *k* = 8, *p* = .007, *I*² = 80.303) between risk appraisals and intention to vaccinate. Six studies reported this relationship, consisting of eight interventions. Forest plots for risk, intention, behaviour, susceptibility, severity, and the relationship between risk and intention can be found in Table S4.

The most common BCT, unique to the intervention condition, was ‘Information about Health Consequences’, which was included in interventions reported by 13 of the included interventions. Other BCTs included Credible Source (*k* = 5) and Information about Social and Environmental Consequences (*k* = 6). On the whole, there were very few unique BCTs used within interventions compared to controls. Three studies had no unique BCTs in the intervention condition compared to the control condition (De Wit, Das, Das, & Vet, 2008; Frew *et al*., 2014 and Godinho *et al*., 2016).

### Study risk of bias

Of the 18 studies included in the review, three had a moderate risk of bias (Bennett *et al*., 2015; Hopfer, 2009 and Vet, de Wit, & Das, 2011), and 15 had a high risk of bias (Higgins & Green, 2011). Plots of the risk of bias assessment per domain and by study can be found in Figure S1. The domain contributing most frequently to an overall high risk of bias rating was ‘Random Sequence Generation’ (unclear descriptions of how participants were randomized to conditions were often not specified, resulting in a rating of ‘unclear’) and ‘Selective Reporting’ (protocols were often unavailable or not mentioned, so there was insufficient information to establish whether all of the intended outcomes had been reported).

### Assessment of heterogeneity

Considerable heterogeneity was present in measures of risk appraisal, *I*² = 76.855; intention, *I*² = 72.613; and behaviour, *I*² = 79.468. As substantial heterogeneity was present, a random‐effects model was used.

### Publication bias

There was evidence of publication bias for the outcome variable behaviour. Trim‐and‐fill analysis made two adjustments, and no change in behaviour was observed (adjusted values can be found in Table S5). There was no evidence of publication bias for the outcomes of risk or intention, and therefore, no adjustments were made.

### Meta‐regression results

All meta‐regression results are given in Table 2.

**Table 2:** Effects of risk appraisals, intention, and behaviours, according to potential moderators

Outcome variable	Moderator	Subgroup	Number of studies/total sample size	*d*	Δ*d*	*Q*	Standard error	Confidence intervals (95%)	Reference group	Number of studies/total sample size (of reference group)	*d* (of reference group)
Risk	Efficacy appraisal also increased	Increased	3/449	0.372	0.242	0.92	0.253	−0.254, 0.738	Not increased	14/6,584	0.130
	Type of risk question used	Conditional question	4/1,083	0.019	−0.218	1.61	0.172	−0.554, 0.119	Unconditional question	12/5,950	0.237
	Illness type	Flu	9/5,023	0.228	−0.122	0.57	0.162	−0.439, 0.196	Other	8/2,125	0.106
		HPV	3/1,490	0.049	0.139	0.45	0.207	−0.207, 0.545	Other	13/5,543	0.188
	Age group	Adult	10/2,105	0.250	−0.239	1.92	0.174	−0.577, 0.099	Other	6/4,928	0.011
		Older adult	5/4,177	−0.000	0.245	1.94	0.175	−0.099, 0.589	Other	11/2,856	0.244
	Pregnancy	Pregnant	3/645	0.396	0.269	1.19	0.247	−0.215, 0.752	Not pregnant	13/6,395	0.127
	BCT information about health Consequences	Included	6/3,449	0.033	−0.238	2.02	0.168	−0.567, 0.090	Not included	10/3,584	0.271
	BCT information about social and environmental consequences	Included	3/694	−0.179	−0.431*	4.58	0.201	−0.826, −0.036	Not included	13/6,339	0.252
	BCT credible source	Included	2/561	0.005							
		Not included	14/6,472	0.204							
	Number of BCTs used	Less than two	10/5,137	0.344	−0.431**	8.25	0.150	−0.726, −0.137	Two or more	6/1,896	−0.088
	Mode of delivery	Digital	8/5,123	0.243	−0.201	1.54	0.162	−0.517, 0.116	Other	8/1,910	0.042
		Human	3/956	−0.252	−0.514**	7.21	0.191	0.139, 0.890	Other	13/6,077	0.262
		Printed Materials	5/954	0.319	−0.201	0.98	0.203	0.560, 0.198	Other	11/6,079	0.118
Intention	Illness type	Flu	8/4,602	0.152	0.034	0.02	0.220	−0.396, 0.465	Other	4/520	0.117
	Age group	Adults	8/1,366	0.112	0.078	0.10	0.246	−0.404, 0.559	Other	4/3,909	0.190
	Pregnancy	Pregnant	3/645	0.045	−0.110	0.14	0.289	−0.675, 0.456	Not pregnant	9/4,630	0.155
	BCT Information about Health Consequences	Included	4/3,047	0.128	−0.007	0.00	0.247	−0.491, 0.477	Not included	8/2,228	0.135
	BCT credible source	Included	1/158	0.062							
		Not included	11/5,117	0.140							
	Number of BCTs used	Less than two	10/4,984	0.103							
		Two or more	2/291	0.372							
	Mode of delivery	Digital	6/4,384	0.126	0.052	0.01	0.230	−0.426, 0.476	Other	6/684	0.151
Behaviour	Illness type	Flu	1/115	0.375							
		HPV	3/1,490	−0.333							
		Pneumonia	1/115	2.000							
	Age group	Adolescent	1/751	−0.045							
		Adult	2/739	−0.482							
		Older adult	1/115	0.871							
	BCT information about health consequences	Included	3/1,116	0.081							
		Not included	1/489	−0.033							
	BCT credible source	Included	2/1,001	−0.471							
		Not included	2/604	0.605							
	BCT information about social and environmental consequences	Included	2/604	0.605							
		Not included	2/1,001	−0.471							
	Mode of delivery	Digital	2/1,200	−0.487							
		Human	2/866	0.589							

*Notes*

Blank cells indicate that there was insufficient variability in the moderator to conduct the analysis (<3 studies).

*p < .05; **p < .01

#### Efficacy appraisals

Efficacy appraisals had no significant association with risk (∆*d* = 0.242, *Q* = 0.92, *p* = .339). Interventions that included efficacy had a higher effect size (*d* = 0.372, *k* = 3) than interventions that did not (*d* = 0.130, *k* = 14).

#### Type of risk question used

The type of risk question used (conditional or unconditional) had no significant association with risk (∆*d* = −0.218, *Q* = 1.61, *p* = .205). Interventions that used unconditional questions had a higher effect on risk (*d* = 0.237, *k* = 12) than interventions that used conditional questions (*d* = 0.019, *k* = 4).

#### Illness type: flu

Illness type had no significant association with risk when flu was the illness being vaccinated against (∆*d* = −0,122, *Q* = 0.57, *p* = 452). Interventions for flu vaccination had a higher effect on risk (*d* = 0.228, *k* = 9) than when interventions were for other illnesses (*d* = 0.106, *k* = 8).

Illness type had no significant association with intention when flu was the illness being vaccinated against (∆*d* = 0.034, *Q* = 0.02, *p* = .876). Interventions for flu vaccination had a higher effect on risk (*d* = 0.152, *k* = 8) than when interventions were for other illnesses (*d* = 0.117, *k* = 4).

#### HPV

Illness type had no significant association with risk when HPV was the illness being vaccinated against (∆*d* = 0.139, *Q* = 0.45, *p* = .500). Interventions for HPV vaccination had a lower effect on risk (*d* = 0.049, *k* = 3) than when interventions were for other illnesses (*d* = 0.188, *k* = 13).

#### Age group: adult

Age group of participants had no significant association with risk when participants were adults (∆*d* = −0.239, *Q* = 1.92, *p* = .166). Interventions had a higher effect on risk when participants were adults (*d* = 0.250, *k* = 10) than when they were other age groups (*d* = 0.011, *k* = 6).

Age group of participants had no significant association with intention when participants were adults (∆*d* = 0.078, *Q* = 0.10, *p* = .751). Interventions had a lower effect on intention when participants were adults (*d* = 0.112, *k* = 80) than when they were other age groups (*d* = 0.190, *k* = 4).

#### Older adult

Age group of participants had no significant association with risk when participants were older adults (∆*d* = 0.245, *Q* = 1.94, *p* = .163). Interventions had a higher effect on risk when participants were other age groups (*d* = 0.244, *k* = 11) than when they were older adults (*d* = −0.000, *k* = 5).

#### Pregnancy

Whether participants were pregnant had no significant association with risk (∆*d* = 0.269, *Q* = 1.19, *p* = .276). Interventions had a higher effect on risk when participants were pregnant (*d* = 0.396, *k* = 3) than when they were not pregnant (*d* = 0.127, *k* = 13).

Whether participants were pregnant had no significant association with intention (∆*d* = −0.110, *Q* = 0.14, *p* = .704). Interventions had a lower effect on intention when participants were pregnant (*d* = 0.045, *k* = 3) than when they were not pregnant (*d* = 0.155, *k* = 9).

#### BCTs: information about health consequences

Including the BCT Information about Health Consequences had no significant association with risk (∆*d* = −0.238, *Q* = 2.02, *p* = .155). Interventions that included Information about Health Consequences had a lower effect on risk (*d* = 0.033, *k* = 6) than interventions that did not (*d* = 0.271, *k* = 10).

Including the BCT Information about Health Consequences had no significant association with intention (∆*d* = −0.007, *Q* = 0.00, *p* = .970). Interventions that included Information about Health Consequences had a lower effect on intention (*d* = 0.128, *k* = 40) than interventions that did not (*d* = 0.135, *k* = 8).

#### Information about social and environmental consequences

Including the BCT Information about Social and Environmental Consequences had a small, significant negative association with risk (Δ*d* = −0.431, *Q *=* *4.58, *p* = .032*). Interventions with this BCT had a lower effect size (*d* = −0.179, *k* = 3) than interventions without this BCT (*d* = 0.252, *k* = 13).

#### Number of BCTs in intervention (less than two, or two or more)

The number of BCTs had a significant negative association with risk (Δ*d* = −0.431, *Q *=* *8.25, *p* = .0004**). Interventions with less than two BCTs had a higher effect size (*d* = 0.344, *k* = 10) than interventions with two or more BCTs (*d* = −0.088, *k* = 6).

#### Mode of delivery: digital

Digital methods of delivery had no significant association with risk (∆*d* = −0.201, *Q* = 1.54, *p* = .215). Interventions that used a digital mode of delivery had a higher effect on risk (*d* = 0.243, *k* = 8) than other modes of delivery (*d* = 0.042, *k* = 8).

Digital methods of delivery had no significant association with intention (∆*d* = 0.052, *Q* = 0.01, *p* = .913). Interventions that used a digital mode of delivery had a lower effect on intention (*d* = 0.126, *k* = 6) than other modes of delivery (*d* = 0.151, *k* = 6).

#### Human

The mode of delivery had a small significant association with risk (∆*d* = 0.514, *Q* = 7.21, *p* = .007**). Interventions delivered by humans had a significantly larger negative effect on risk (*d* = −0.252, *k* = 3) compared to those where other methods of delivery were used (*d* = 0.262, *k* = 13).

#### Printed material

Printed materials had no significant association with risk (∆*d* = −0.201, *Q* = 0.98, *p* = .323). Interventions that used printed materials had a higher effect on risk (*d* = 0.319, *k* = 5) than other modes of delivery (*d* = 0.118, *k* = 11).

Where subgroups within a moderator contained insufficient studies (e.g., for illness type within studies measuring intention, there were only two studies that examined hepatitis B and two that examined HPV), but there was at least one reference group with three or more studies (e.g., flu had eight studies), the other subgroups were combined (e.g., hepatitis and HPV combined to create an ‘other illness category’) and compared to the reference group (e.g., flu).

## Discussion

### Principal findings

Overall, whilst interventions containing risk messages did not increase intention to vaccinate or vaccination behaviour, they did have a small effect on risk appraisal. There was a small relationship between vaccination risk appraisal and intention to vaccinate. There was a small but significant pooled effect of interventions on susceptibility, but no pooled effect on severity. Interventions with higher numbers of BCTs and those delivered in person (as opposed to via digital or printed material) had smaller effects on risk appraisals. The majority of studies had high risk of bias, often due to multiple indicators being unclear.

Interventions in the present review were found to include few BCTs, with the most commonly used being Information about Consequences, Credible Source, and Information about Social and Environmental Consequences. The presence of Information about Social and Environmental Consequences had a negative effect on vaccination risk appraisal, suggesting that the presence of this BCT within interventions reduced individuals’ appraisals of risk. Interestingly, of the three studies that included this BCT, only one successfully increased efficacy appraisal. It is possible therefore that this finding reflects an element of defensive processing (see Wright, 2010). In other words, intervention content that triggers individuals to appraise the risk of illness without also ensuring that they feel able to perform a behaviour perceived as effective may lead them to adopt coping strategies such as denial or avoidance.

Meta‐regression analysis showed that the number of BCTs included in an intervention had a small, significant negative effect on risk. Specifically, interventions that had three or more unique BCTs decreased risk appraisal. This unexpected finding is in contrast to other reviews which have found that including more BCTs has a greater effect on behaviour change (Cradock *et al*., 2017; Webb, Joseph, Yardley, & Michie, 2010). One possible explanation for this may be that brief information on vaccination is preferable. Shorter, more concise material may increase engagement, and therefore may be more effective in increasing risk appraisal.

Meta‐regression analysis also showed that there was a difference in the effect of interventions delivered by people, compared to those delivered digitally or using printed material. Specifically, those delivered by people had a negative effect on risk (whilst interventions delivered digitally or with printed materials had a positive effect). This may be explained in a number of ways; firstly, research suggests that risk information is often communicated less effectively when done so verbally. Furthermore, interventions delivered face‐to‐face may be more at risk of variation in the way they are delivered, compared to more standardized paper digital materials. Finally, some medical professionals may demonstrate a preference to promote informed choices of individuals, thus tempering messages that actively promote vaccination uptake (French & Marteau, 2007).

### Strengths and weaknesses

Review‐level strengths include that the present review was conducted and reported in line with PRISMA guidelines (completed PRISMA checklist can be found in Supporting Information Table S6) and the Meta‐Analysis Reporting Methods (MARS, 2008). Stringent inclusion criteria ensured that only studies that could contribute to understanding about the impact of interventions on risk appraisal on vaccination intention or uptake were included. This however also introduced a weakness in the ability of the review to draw conclusions, in that few studies met the inclusion criteria and could therefore be included in the review. This indicates the paucity of experimental studies that exist in this field and the need for more to further increase knowledge in this area. Grey literature was searched for and included, so the authors are confident that all appropriate studies were found and included in the review. However, due to limited resources, only studies in the English language were included in the review. This may have excluded other potentially useful contributions to the topic.

A strength of the present systematic review is the thorough risk of bias assessment it was subject to, using the Cochrane Risk of Bias Assessment Tool, which identified the frequent unclear reporting leading to unclear risk of bias assessments.

Study‐level weaknesses include that the majority of studies were conducted in the United States. International differences in health care systems and vaccination programmes may mean that studies conducted in the United States may not be generalizable to populations within the United Kingdom or other European countries, nor to low‐ to middle‐income countries. A further weakness lies with the failure of most studies to measure vaccination behaviour, with studies largely measuring intention to vaccinate instead.

The illness being vaccinated against varied greatly amongst studies in this review. There is the potential that differences in appraisals of risk may exist between illnesses, meaning that the effect of risk on vaccination differs accordingly. For example, appraisals of hepatitis B risk may be higher than for influenza risk due to the belief that the former causes serious liver damage, whereas the latter has few serious consequences. This means it is potentially problematic to directly compare interventions, as different risk appraisal processes may be present. Equally, how common an illness is may influence the success of the intervention, as less common illnesses may be perceived as more threatening and associated with higher appraisals of risk. In addition, some illnesses examined in the included studies required one dose of vaccine (such as flu), whereas other illnesses (such as HPV) required up to three doses. These behaviours are not directly comparable, with the latter being more difficult to perform. There were too few studies in the present review to compare the effect of risk appraisal on vaccination behaviour according to illness type or frequency of doses. Meta‐regression was often not possible due to there being insufficient studies in each subgroup, again highlighting the need for additional experimental studies in this field.

One strength of the included studies themselves was the use of composite measures of risk rather than single measures of risk, which was coded in 12 of the 18 included studies. Risk is a complex construct, which is better measured using composite measures due to the increased validity of multiple measures (van der Velde *et al*., 1996).

A further strength of the included studies is the study setting. Of the 18 included studies, 17 were conducted in a community rather than a laboratory setting. This is advantageous as it reduces the chance of bias as a result of artificial settings, and reflects real behavioural decisions, rather than a hypothetical decision.

The present review highlighted a number of weaknesses in the existing literature on risk appraisal and vaccination uptake. First, the majority of included studies were rated as demonstrating an overall high risk of bias, largely attributable to the fact that a large proportion of domains across all studies were rated as ‘unclear’. A rating of unclear reflects limitations in the reporting of the study rather than necessarily being a weakness in methodology. However, a high risk of bias suggests that it is unclear whether results of the study reflect a true effect of the intervention, and therefore, a degree of caution should be employed when interpreting the results. The presence of high risk of bias ratings reduces confidence in the findings and makes it difficult to conclude whether interventions that include risk messages are indeed successful in increasing risk appraisal or the uptake of vaccination. Once again, this leads to calls for better conducted and reported studies on this topic.

Second, it should be noted that in a number of the included studies, a similar level of intervention content was delivered in the control groups, as in the intervention groups. One explanation for this may be that detailed intervention descriptions were often unavailable in the papers and contact with authors for further details was met with limited response. Therefore, BCT coding was often only possible on the information within the paper itself, and it is acknowledged that full interventions may have included more BCTs in their entirety.

The BCT ‘Information about Health Consequences’ was coded within the control group of six included studies. Whilst only BCTs unique to the intervention group were included when examining the moderating effect of BCTs, the presence of BCTs within control groups that would be expected to have an impact of risk appraisal means that the relationship between risk and vaccination behaviour may be underestimated by our analysis. It is also important to examine the dose of BCTs in both the intervention and control groups, as although a BCT may be present in both (and therefore not coded as a BCT unique to the intervention condition), it may appear more frequently, or may be a stronger influence in the intervention condition, than in the control condition (this can be seen in the practical application table in Table S1 where BCT and dose of both intervention and control condition are detailed for each included study). This is supported by previous findings that intervention effects can be reduced in situations where the level of care received by the control group is higher (de Bruin *et al*., 2010). Furthermore, only including those BCTs that are unique to the intervention group may mean that clusters of BCTs working together to change behaviour may be ignored.

It is important to consider that the primary aim of the included studies was often not to examine the effectiveness of an intervention involving a risk message, and so the interventions were often not specifically aiming to increase risk appraisal alone. The decision to include all interventions that targeted risk, regardless of whether they also targeted a change in other variables, means that the effect of interventions on intentions and behaviour is confounded. The overall number of studies included in the review was too small to enable a number of planed analyses to be performed, and therefore, requiring included studies to only be examining risk appraisal would have reduced the pool further. Consequently, there is a need for more studies which aim to manipulate risk and efficacy exclusively (ideally with factorial design so that the independent and interaction effects of each can be examined).

Also, the studies often tested methods of delivery, for example, examining the effect of gain versus loss framing of risk information. Increases in risk appraisal found in included studies may therefore be attributable to other factors that are unrelated to the content of the intervention.

Finally, limitations exist relating to how risk was measured. For example, not all included studies measured levels of risk pre‐intervention. This makes it unclear whether differences in risk between conditions existed at baseline, thus influencing differences between conditions post‐interventions. Furthermore, the majority of studies included in this review measured risk using unconditional risk questions. To correctly assess appraisals of risk, participants should be asked about how likely they are to become ill if they do not have the vaccination. By asking unconditional questions, participants may be taking into account their good intention. In this situation, risk appraisals are based on the perceived likelihood of becoming ill after having the vaccination, rather than the likelihood of becoming ill without it (Weinstein *et al*., 1998). This makes it difficult to draw firm conclusions about the influence that risk messages have on risk appraisal and vaccination uptake. Finally, the way risk was measured varied greatly between studies, with some measuring risk in terms of likelihood, some measuring severity, and some measuring both likelihood and severity. It is acknowledged that these ways of measuring risk are theoretically different and depending on the measurement choices made may have impacted upon the ability of studies to capture any intervention effects.

### What this study adds

This is the first systematic review to examine the effect of interventions on risk appraisal and vaccination intentions or uptake using only experimental studies. It builds on a previous meta‐analysis in this area (Brewer *et al*., 2007) which included not only experimental studies, but also prospective and cross‐sectional studies. Including only experimental studies is important because it increases the strength of conclusions which can be drawn about the effect of interventions on risk and behaviour. The findings of this review are however inconclusive. The lack of unique BCT content within intervention conditions, along with the high risk of bias and almost total reliance on unconditional measures of risk by studies examining those interventions, means that we cannot be confident in the findings. Consequently, the potential value of this type of review in better understanding how to increase risk in order to increase vaccination behaviour is lost. Instead, its value is in shining a light on the paucity of experimental studies in this area, and the quality of methods and reporting used. It should be noted that eight of the 18 included studies were conducted in the past 5 years. This is encouraging as it indicates increasing use of experimental designs.

A secondary aim of the present review was to examine the relationship between risk and vaccination intention and uptake. Earlier work by Sheeran and colleagues found that risk appraisal had a small but significant effect on vaccination intention (*d* = 0.38) and behaviour (*d* = 0.33). Whilst the review by Sheeran and colleagues only included studies that had a significant effect on susceptibility or severity in order to enable this relationship to be observed (pooled effects being *d* = 0.75 and *d* = 0.56, respectively), the inclusion of all studies in the present review regardless of their success in changing risk appraisal reduced the size of the overall effect. Given the small pooled effect on risk appraisal, the possible reasons for which have been discussed above, it is unsurprising then that no relationship between risk and vaccination intentions or uptake was observed. The present review is therefore unable to contribute new knowledge about the relationship between risk and vaccination intentions or uptake.

This systematic review builds on work conducted by Sheeran *et al*. (2014) as it adds to evidence more broadly about the relationship between risk appraisal and behaviour. The current review included studies that would have been omitted by Sheeran and colleagues which only included RCTs that were successful in changing risk appraisals. Restricting studies to those examining single health behaviour controls for factors relating to the nature of the behaviour itself which may confound results.

### Implications for practice

The present review demonstrates that interventions in included studies utilize relatively few BCTs. For this reason, specific recommendations regarding which BCTs should be included in interventions to successfully increase vaccination intention or uptake cannot be made. There is compelling evidence that providing information about the risk of health or the risk of failing to carry out the health behaviour alone is not sufficient to elicit behaviour change (French, Cameron, Benton, Deaton, & Harvie, 2017). Additional BCTs may improve the effectiveness of interventions in increasing risk appraisal and subsequent uptake of vaccination.

Recent research suggests that simultaneously increasing efficacy appraisals with risk appraisals is an important parameter for having an overall effect on behaviour. Evidence suggests that the effect of increasing risk appraisal on intention or behaviour is further increased when efficacy appraisals are also high (Kok *et al*., 2015; Sheeran *et al*., 2014). Unfortunately, because only three studies within this review significantly increased efficacy appraisals, conclusions could not be drawn about the interaction between risk appraisals and efficacy appraisals. This highlights the need for future research to examine the effect of increasing both risk and efficacy appraisals, ideally using full factorial designs that enable individual and interaction effects to be observed. In the meantime, interventions should aim to target an increase in self‐efficacy and response efficacy simultaneously with risk appraisal in order to prevent defensive processing. The present review found that interventions delivered by people, as opposed to those delivered digitally or via printed materials, were less effective at increasing risk appraisals. This may be because risk information communicated verbally is more difficult to absorb and understand. This concurs with other work which has found that interventions utilizing images or visual components have been found to be successful predictors of changing risk appraisal (French *et al*., 2017). Accordingly, it is advised that future interventions aiming to communicate risk incorporate images into their design.

### Implications for research

The present review highlights the need for robust, well‐reported experimental studies examining the effect of interventions on risk and vaccination behaviour. Reporting of methods by included studies was often vague and incomplete, and future studies would benefit from clearer more transparent reporting. As previously highlighted, the reporting of methods and intervention content by authors is currently inadequate. This makes assessing the quality of experimental studies and their risk of bias and accurately coding the presence of BCTs difficult. We acknowledge that journal restrictions may prevent detailed reporting of intervention content within the paper itself. As an alternative, we urge authors to use [Link], [Link], [Link], [Link], [Link], [Link], [Link] where permitted, to publish intervention content separately, or to make content descriptions available via the Web.

Risk of bias assessment revealed that the main potential source of bias was ‘Random Sequence Generation’, and of the 18 studies assessed, eight were allocated an unclear rating and three a high rating. In addition to this, 13 studies were allocated an unclear rating for ‘Selective Reporting’, reflecting a need for better reporting.

Future research would benefit from exploring potential reasons why interventions using digital or printed methods may be more effective in increasing risk appraisals, than those delivered face‐to‐face. This may include difficulties communicating risk verbally, and the reluctance of medical professionals to actively recommend vaccination. Furthermore, it would be beneficial for future research to explore whether briefer interventions are more successful in increasing risk appraisal than longer, more in‐depth interventions.

### Conclusion

This systematic review is the first to explore the influence that interventions containing risk messages have on risk appraisal and vaccination intention and uptake using only experimental studies. Weaknesses in the included studies mean that it is not possible to draw firm conclusions about effect of interventions on risk, nor to examine the relationship between risk appraisal and vaccination behaviour. Successful interventions might benefit from using more BCTs, and from targeting increases in self‐efficacy and response efficacy, in addition to risk appraisal.

## Funding

Funding for this review came from a PhD Studentship from Coventry University. No other funding was obtained.

## Conflict of interest

All authors declare no conflict of interest.

## Supporting information


**Appendix S1.** Search terms used in database searches.

**Figure S1.** Risk of bias figures.

**Table S1.** Practical Applications, dose and mode of delivery.

**Table S2.** Summary table of characteristics of included studies.

**Table S3.** Effect sizes of included studies, and BCTs in included studies.

**Table S4.** Forest plots of outcome variables.

**Table S5.** Trim and fill adjusted values.

**Table S6.** PRISMA 2009 checklist.

**Table S7.** Raw data file; Relationship intention and risk.

**Table S8.** Raw data file; Severity.

**Table S9.** Raw data file; Susceptibility.

**Table S10.** Raw data file; Behaviour.

**Table S11.** Raw data file; Intention.

**Table S12.** Raw data file; Risk.
