# Supplementary material for: Do interventions containing risk messages increase risk appraisal and the subsequent vaccination intentions and uptake? – A systematic review and meta‐analysis
Source: Br J Health Psychol. 2018 Sep 17;23(4):1084–106. doi: 10.1111/bjhp.12340 (PMC6767484; doi:10.1111/bjhp.12340)
Supplement: Supplementary file 1 — Appendix S1. Search terms used in database searches. [file BJHP-23-1084-s001.docx]

Supplemental material 1:

Search terms used in database searches

Search terms were based on those used by Sheeran, Harris and Epton (2014). Additional terms were included to identify articles examining fear appeals, and to identify articles relating to vaccination behaviour specifically.

The first filter, for study design used the terms

experiment OR randomized OR randomised OR controlled OR trial OR manipulated OR evaluation OR program*.

The second filter to capture outcome measures used the search terms intention* OR behav* OR action OR acceptance OR motivation OR performance.

The third filter; risk appraisals was searched for using the terms risk perception OR perceived risk OR risk appraisal OR risk judgment OR perceived susceptibility OR perceived vulnerability OR perceived likelihood OR comparative optimism OR unrealistic optimism OR optimistic bias OR risk message OR risk communication OR scare tactic OR shock tactic OR fear OR appeal OR persuasion OR campaign OR perceived threat OR perceived severity OR Protection Motivation Theory OR Health Belief Model OR Precaution Adoption Process OR Health Action Process Approach OR Parallel Process Model OR Extended Parallel Process Model.

The fourth filter, for behaviour used the search terms vaccin* OR immun* OR inoculation* OR shot .
