# Supplementary material for: Do interventions containing risk messages increase risk appraisal and the subsequent vaccination intentions and uptake? – A systematic review and meta‐analysis
Source: Br J Health Psychol. 2018 Sep 17;23(4):1084–106. doi: 10.1111/bjhp.12340 (PMC6767484; doi:10.1111/bjhp.12340)
Supplement: Supplementary file 2 — Figure S1. Risk of bias figures. [file BJHP-23-1084-s002.docx]

Supplemental material 6:

Risk of bias figures

Risk of bias ratings per domain


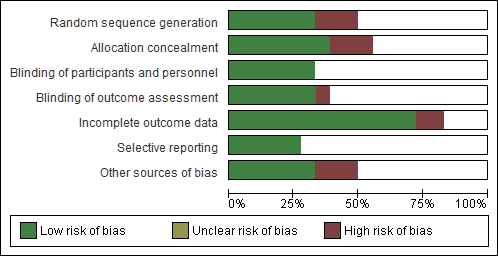


Black = High risk of bias, White= Unclear risk of bias, Grey= Low risk of bias

Risk of bias ratings by study


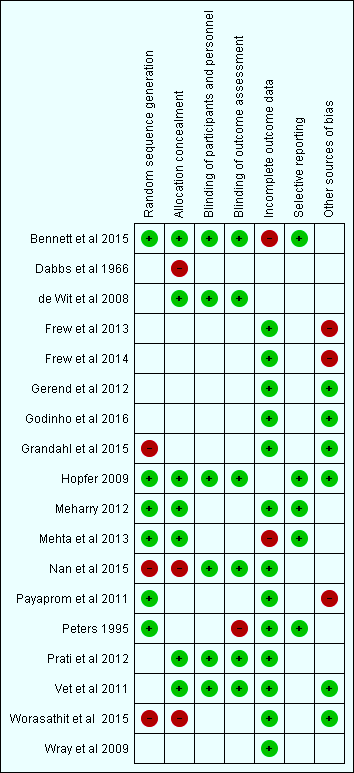


(Black= High risk of bias, Blank= Unclear risk of bias, Grey= Low risk of bias)
