# Supplementary material for: Do interventions containing risk messages increase risk appraisal and the subsequent vaccination intentions and uptake? – A systematic review and meta‐analysis
Source: Br J Health Psychol. 2018 Sep 17;23(4):1084–106. doi: 10.1111/bjhp.12340 (PMC6767484; doi:10.1111/bjhp.12340)
Supplement: Supplementary file 3 — Table S1. Practical Applications, dose and mode of delivery. [file BJHP-23-1084-s003.docx]

| Author/ year | Condition (or combination of conditions as received by participants) | Mode of delivery | BCTs | Practical Applications | Dose (number of times the BCT appears in the description of the intervention) |
| --- | --- | --- | --- | --- | --- |
| Bennett, Patel, Carlos, Zochowski, Pennewell, Chi and Dalton (2015) | Control | Digital; Computer; Website. | No BCTs; information about vaccine only | n/a | 0 |
|  | Intervention (MeFirst website) | Digital; Computer; Website. | Information on health consequences. | Factual information on HPV and the HPV vaccine given through topic tailored website, including statistics of incidence. | 7 (Info says 7 tailored webpages. Only explained in summary form) |
| Dabbs and Leventhal (1966)  (3x2x2 factorial design) | Control (no fear), high pain, high effectiveness | Printed material; Leaflet. | Information about health consequences  Information about Emotional Consequences | The pamphlet provided information about the vaccination being painful  Participants were warned that they would feel pain as a result of performing the behaviour. | 1  1 |
|  | Control (no fear), high pain, low effectiveness | Printed material; Leaflet. | Information about health consequences  Information about Emotional Consequences | The pamphlet provided information about the vaccination being painful  Participants were warned that they would feel pain as a result of performing the behaviour. | 1  1 |
|  | Control (no fear), low pain, low effectiveness | Printed material; Leaflet. | No BCTs Identified | n/a | 0 |
|  | Control (no fear), low pain, high effectiveness | Printed material; Leaflet. | No BCTs Identified | n/a | 0 |
|  | Low fear, high pain, high effectiveness | Printed material; Leaflet. | Information about health consequences  Information about Emotional Consequences | The pamphlet provided information about the symptoms of tetanus including a case history  The pamphlet provided information about the vaccination being painful  Participants were warned that they would feel pain as a result of performing the behaviour. | 3  1  1 |
|  | Low fear, high pain, low effectiveness | Printed material; Leaflet. | Information about health consequences  Information about Emotional Consequences | The pamphlet provided information about the symptoms of tetanus including a case history  The pamphlet provided information about the vaccination being painful  Participants were warned that they would feel pain as a result of performing the behaviour. | 3  1  1 |
|  | Low fear, low pain, low effectiveness | Printed material; Leaflet. | Information about Health Consequences | The pamphlet provided information about the symptoms of tetanus including a case history | 2 |
|  | Low fear, low pain, high effectiveness | Printed material; Leaflet. | Information about Health Consequences | The pamphlet provided information about the symptoms of tetanus including a case history | 2 |
|  | High fear, high pain, high effectiveness | Printed material; Leaflet. | Information about health consequences  Information about Emotional Consequences  Salience of consequences | The pamphlet provided information about the symptoms of tetanus including a case history  The pamphlet provided information about the vaccination being painful  Participants were warned that they would feel pain as a result of performing the behaviour.  Information is given to high fear conditions about the chances of death as a result of tetanus, using detailed and more emotive language. Colour images are also used in the high fear condition. | 3  1  1  2 |
|  | High fear, high pain, low effectiveness | Printed material; Leaflet. | Information about health consequences  Information about Emotional Consequences  Salience of consequences | The pamphlet provided information about the symptoms of tetanus including a case history  The pamphlet provided information about the vaccination being painful  Participants were warned that they would feel pain as a result of performing the behaviour.  Information is given to high fear conditions about the chances of death as a result of tetanus, using detailed and more emotive language. Colour images are also used in the high fear condition. | 3  1  1  2 |
|  | High fear, low pain, low effectiveness | Printed material; Leaflet. | Information about health consequences  Salience of consequences | The pamphlet provided information about the symptoms of tetanus including a case history  Information is given to high fear conditions about the chances of death as a result of tetanus, using detailed and more emotive language. Colour images are also used in the high fear condition. | 2  2 |
|  | High fear, low pain, high effectiveness | Printed material; Leaflet. | Information about health consequences  Salience of consequences | The pamphlet provided information about the symptoms of tetanus including a case history  Information is given to high fear conditions about the chances of death as a result of tetanus, using detailed and more emotive language. Colour images are also used in the high fear condition. | 2  2 |
| de Wit, Das and Vet (2008) | Control; Brief mention of risk with no evidence | Digital; Computer; Website | Information about health consequences | Introductory statement on the website (for all conditions) discusses the risk factors for men who have sex with men, in being infected with HBV. | 1 |
|  | Control; No risk information | Digital; Computer; Website | Information about health consequences | Introductory statement on the website (for all conditions) discusses the risk factors for men who have sex with men, in being infected with HBV. | 1 |
|  | Intervention; Statistic evidence | Digital; Computer; Website | Information about health consequences | Introductory statement on the website (for all conditions) discusses the risk factors for men who have sex with men, in being infected with HBV. | 5 |
|  | Intervention; Narrative evidence | Digital; Computer; Website | Information about health consequences | Introductory statement on the website (for all conditions) discusses the risk factors for men who have sex with men, in being infected with HBV. | 3 |
| Frew, Owens, Saint-Victor, Benedict, Zhang and Omer (2014) | Control | Printed material; Leaflet. | Information about Health consequences | Information presented in written form to all participants about the flu vaccination, incidence of deaths from flu in pregnancy, and safety of the vaccination in pregnancy. | 1 |
|  | Intervention; Loss framed message | Printed material; Leaflet. | Information about Health consequences | Information presented in written form to all participants about the flu vaccination, incidence of deaths from flu in pregnancy, and safety of the vaccination in pregnancy.  Participants in the loss-framed message also given information about risks to themselves and unborn baby from flu. | 2 |
|  | Intervention; Gain framed message | Printed material; Leaflet. | Information about Health consequences | Information presented in written form to all participants about the flu vaccination, incidence of deaths from flu in pregnancy, and safety of the vaccination in pregnancy. | 1 |
| Frew, Zhang, Saint-Victor, Schade, Benedict, Banan, Ren and Omer (2013) | Control | Printed material; Leaflet. | Information about Health consequences | Information presented to all participants in written form, about the flu vaccination, incidence of deaths from flu in pregnancy, and safety of the vaccination in pregnancy. | 1 |
|  | Intervention; Loss framed message | Printed material; Leaflet. | Information about Health consequences  Salience of consequences | Information presented to all participants in written form, about the flu vaccination, incidence of deaths from flu in pregnancy, and safety of the vaccination in pregnancy.  Participants in the loss-framed message also given information about risks to themselves and unborn baby from flu.  Participants in the loss-framed message were presented with visual image showing ambulance and stretcher saying ‘don’t risk the life of your unborn child by skipping a flu shot’ | 2  1 |
|  | Intervention; Gain framed message | Printed material; Leaflet. | Information about Health consequences | Information presented to all participants in written form, about the flu vaccination, incidence of deaths from flu in pregnancy, and safety of the vaccination in pregnancy. | 1 |
| Gerend and Shepherd (2012) | Control | Digital; Television; Other (video) | Information about health consequences  Salience of consequences | Information is given in the video, about how HPV is caused and spread.  Information is also given about there being no cure for HPV  Information is given about the link between HPV and cancer | 2  1 |
|  | Intervention; Loss framed message | Digital; Television; Other (video) | Information about health consequences  Salience of consequences  Anticipated regret  Information about social and environmental consequences  Information about emotional consequences | Information is given in the video, about how HPV is caused and spread.  Information is also given about there being no cure for HPV  Information is given about the link between HPV and cancer  The video also suggest that not getting vaccinated may result in feelings of regret  The video discusses risks, including the risk of passing HPV to others  The video makes the suggestion that not getting vaccinated may result in feelings of worry. | 3  1  3  1  1  1 |
|  | Intervention; Gain framed message | Digital; Television; Other (video) | Information about Health Consequences | Information is given in the video, about how HPV is caused and spread.  Information is also given about there being no cure for HPV | 1  1 |
| Godinho, Yardley, Marcu, Mowbray, Beard and Michie (2016) | Control; Standard Department of Health message | Digital; Computer; Website | Information about social and environmental consequences  Information about health consequences | The website provides information about how the vaccination will prevent spread to family and friends  The website discusses what some of the consequences of not getting vaccinated may be to health, and that the vaccination will protect from flu | 2  2 |
|  | Intervention; Shortened Department of Health message | Digital; Computer; Website | Information about Health Consequences | The website informs that the vaccination will protect from flu | 1 |
|  | Intervention; Shortened risk-reducing message | Digital; Computer; Website | Information about social and environmental consequences  Information about health consequences | The website provides information about how the vaccination will prevent spread to family and friends  The website discusses how the vaccination will reduce chances of being seriously ill from flu | 1  1 |
|  | Intervention; Shortened health-enhancing | Digital; Computer; Website | Information about Health Consequences | The website discusses some of the consequences of flu | 1 |
| Grandahl, Rosenblad, Stenhammar, Tyden, Westerling, Larsson, Oscarson, Andrae, Dalianis and Neveus (2016) | Control | Human; face-to-face | No BCTs identifiable in information available: Control group received general information. | n/a | 0 |
|  | Intervention; Education | Human; face-to-face | Information about health consequences  Credible Source | The school nurse uses a flipchart and leaflet to discuss information on general facts about the virus and what HPV can cause.  The intervention was delivered face to face by school nurse | 2  1 |
| Hopfer (2009) | Control; no message control | No intervention delivered | No BCTs identified | n/a | 0 |
|  | Control; Video control | Digital; Television; Other (video) | No BCTs identified | n/a | 0 |
|  | Control; Website control | Digital; Computer; Website | No BCTS identified | n/a | 0 |
|  | Intervention; Peer condition | Digital; Computer; Website | Credible Source | Evidence on the website is from CDC, about the vaccination being safe and effective | 1 |
|  | Intervention; Provider condition | Digital; Computer; Website | Credible Source  Information about Health Consequences | Evidence on the website is from CDC, about the vaccination being safe and effective  Information is contained within the website that suggests that some people who were not vaccinated went on to develop cervical cancer | 1  1 |
|  | Intervention; Peer and Provider condition | Digital; Computer; Website | Credible Source | Evidence on the website is from CDC, about the vaccination being safe and effective | 1 |
| Meharry (2012) | Control | No intervention delivered. | No BCTs identified | n/a | 0 |
|  | Intervention; Pamphlet | Printed material; Leaflet | Information about Health Consequences | Pamphlet gives information about symptoms of flu, risks of flu to unborn baby and how the vaccine helps protect pregnant women and unborn babies. | 4 |
|  | Intervention; Pamphlet and Benefit Statement | Printed material; Leaflet | Information about Health Consequences | Pamphlet gives information about symptoms of flu, risks of flu to unborn baby and how the vaccine helps protect pregnant women and unborn babies.  Benefit statement that is read out, discusses that flu vaccine will protect self and baby from flu. | 5 |
| Mehta, Sharma and Lee (2013) | Control | Human; Face-to-face | No BCTs identified | n/a | 0 |
|  | Intervention; Health Belief Model based intervention | Human; Face-to-face | Information about health consequences  Prompts/ cues  Instruction on how to perform the behaviour  Information about social and environmental consequences | Information was given face-to-face to participants, about negative consequences of HPV, and information about impact of vaccination on health.  Individuals were asked to identify ways to remind themselves to be vaccinated.  Participants were given step-by-step instructions on how to get vaccinated.  Participants were given information about protection for others | 2  1  1  1 |
| Nan, Dahlstrom, Richards and Rangarajan (2015) | Control; Statistical information | Digital; Computer; Website | Information about health consequences | All participants received identical first and last paragraphs on the website- information from official reports about HPV | 3 |
|  | Intervention; 1^st^ person narrative | Digital; Computer; Website | Information about Health Consequences | All participants received identical first and last paragraphs on the website- information from official reports about HPV. Experimental conditionals also received information about consequences to women, consequences Information about Health Consequences to men, consequences of the illness. | 5 |
|  | Intervention; 3^rd^ person narrative | Digital; Computer; Website | Information about Health Consequences | All participants received identical first and last paragraphs on the website- information from official reports about HPV. Experimental conditionals also received information about consequences to women, consequences to men, consequences of the illness. | 5 |
|  | Intervention; Hybrid 1^st^ person | Digital; Computer; Website | Information about Health Consequences | All participants received identical first and last paragraphs on the website- information from official reports about HPV. Experimental conditionals also received information about consequences to women, consequences to men, consequences of the illness. | 5 |
|  | Intervention; Hybrid 3^rd^ person | Digital; Computer; Website | Information about Health Consequences | All participants received identical first and last paragraphs on the website- information from official reports about HPV. Experimental conditionals also received information about consequences to women, consequences to men, consequences of the illness. | 5 |
| Payaprom, Bennett, Alabaster and Tantipong (2011) | Control; Usual practice | Printed material; Leaflet | Information about health consequences | Standard leaflet contained information about symptoms of flu and information about the vaccine | 1 |
|  | Intervention; Health Action Process intervention | Printed material; Leaflet | Information about health consequences  Information about other’s approval  Action planning  Goal Setting (Behaviour) | Intervention group received information in a leaflet about susceptibility to flu and potential complications.  Accounts from people who had had the vaccination, were provided in the leaflet, to increase normative beliefs around vaccination.  Participants were encouraged to set a specific goal, and write a statement of intent with detail of where, how etc.  Participants were encouraged to set a goal of having the vaccination, and to plan it thoroughly. | 1  1  1  1 |
| Peters (1995) | Control; pre and post test | No intervention delivered | No BCTs identified | n/a | 0 |
|  | Control; post test | No intervention delivered | No BCTs identified | n/a | 0 |
|  | Intervention; Experimental pre and post test | Human; Face-to-face | Information about health consequences  Information about social and environmental consequences | Intervention pamphlet provides information about risks of flu to health, and how the vaccine can prevent illness.  Intervention pamphlet discusses the risk of passing flu to other people, and that the vaccine can prevent the spread to others. | 2  2 |
|  | Intervention; Experimental post test | Human; Face-to-face | Information about health consequences  Information about social and environmental consequences | Intervention pamphlet provides information about risks of flu to health, and how the vaccine can prevent illness.  Intervention pamphlet discusses the risk of passing flu to other people, and that the vaccine can prevent the spread to others. | 2  2 |
| Prati, Pietrantoni and Zani (2012) | Control; no message | No Intervention delivered | No BCTs identified | n/a | 0 |
|  | Intervention; | Digital; Computer; Website | Credible Source | Messages on the website were formatted to look like a mass media campaign by Italian Minister of Health | 1 |
|  | Intervention; | Digital; Computer; Website | Credible Source | Messages on the website were formatted to look like a mass media campaign by Italian Minister of Health | 1 |
| Vet, de Wit and Das (2011)  (2x2 factorial design) | Control; No communication | No intervention delivered | No BCTS identified | n/a | 0 |
|  | Intervention; Risk communication | Digital; Computer; Website | Information about Health Consequences | Information was contained in the website, about the vaccination being the only way to protect self from Hepatitis. | 1 |
|  | Intervention; Social norm communication | Digital; Computer; Website | Information about other’s approval | Messages in the website discusses fear of negative reactions about Hepatitis from others. | 2 |
|  | Intervention; Combined risk and social norm communication | Digital; Computer; Website | Information about Health Consequences  Information about other’s approval | Information was contained in the website, about the vaccination being the only way to protect self from Hepatitis.  Messages in the website discusses fear of negative reactions about Hepatitis from others. | 1  2 |
| Worasathit, Waltana, Okanurak, Songthap, Dhitavat and Pitisuttithum (2015) | Control; no intervention | No intervention delivered | No BCTs identified | n/a | 0 |
|  | Intervention; Educational intervention | Digital; Television; Other (Video) | Information about health consequences | Information is contained in the educational video regarding flu symptoms and complications, and impact of vaccination on prevention. | 2  N.B. Can’t code dose accurately as full intervention not available |
| Wray, Buskirk, Jupka, Lapka, Jacobsen, Pakpahan, Gary and Wortley (2009) | Control; Vaccine information statement | Printed materials; Leaflet | Information about health consequences | Information is present in the leaflet, about how the vaccination can protect from illness from flu, and how it won’t make you ill. | 2 |
|  | Intervention; Vaccine Safety message | Printed materials; Leaflet | Information about health consequences  Salience of consequences | Information is present in the leaflet, about illness as a result of flu, and how vaccination itself won’t make you ill.  The leaflet contains information about serious complications as a result of getting the flu, and rates of death as a result of flu. | 2  1 |
