# Supplementary material for: Do interventions containing risk messages increase risk appraisal and the subsequent vaccination intentions and uptake? – A systematic review and meta‐analysis
Source: Br J Health Psychol. 2018 Sep 17;23(4):1084–106. doi: 10.1111/bjhp.12340 (PMC6767484; doi:10.1111/bjhp.12340)
Supplement: Supplementary file 4 — Table S2. Summary table of characteristics of included studies. [file BJHP-23-1084-s004.docx]

Supplemental material 3:

Summary table of characteristics of included studies

| Lead author/ year | Total sample size | Study conditions | Mode of intervention delivery | % female | Mean age | Study country (and high or medium/ low income country) | Illness being vaccinated against | Participants pregnant or not | Composite or single measure of risk? |  |
| --- | --- | --- | --- | --- | --- | --- | --- | --- | --- | --- |
| Bennett et al (2015) | 661 | Experimental MeFirst condition (n 330) and control condition (n 331). | Digital; Computer | 100% | 21 | US  (High income) | HPV | No | Single | |
| Dabbs et al (1966) | 182 | Fear (low fear, high fear and no fear), Effectiveness (low effectiveness and high effectiveness) and Pain (low pain and high pain).  No sample sizes for each condition was provided. | Printed material | Not specified | Not specified (college seniors) | US  (High income) | Tetanus | Not | Composite | |
| De Wit et al (2008) | 118 | Narrative evidence (n 24), Statistical evidence (n 26), No evidence control (n 38) and mere risk assertion (n 30). | Digital; Computer | 0% | 38.3 | Netherlands (High income) | Hepatitis B | No | Composite | |
|  |  |  |  |  |  |  |  |  |  | |
| Frew et al (2014) | 251 | Gain-framed (n 85), loss-framed (n 87) and control conditions (n 79). | Printed material | 100% | Age range 18-45 | US  (High income) | Flu | Yes | Single | |
| Frew et al (2013) | 261 | Gain-framed (n 87), loss-framed (n 90) and control conditions (n 90) | Printed material | 100% | 25.8 | US  (High income) | Flu | Yes | Single | |
| Gerend et al (2012) | 739 | Gain-framed (n 250), loss-framed (n 243) and control conditions (n 246). | Digital; Television | 100% | 21 | US  (High income) | HPV | No | Composite | |
| Godinho et al 2016 | 1424 | Standard DoH message (n 356), shortened DoH (n 356), shortened risk-reducing message (n 356) and shortened health- enhancing message (n 356). | Digital; Computer | 50.3% | Largest group 55-75 | UK (High income) | Flu | No | Composite | |
| Grandahl et al (2015) | 751 | Education intervention condition (n 394) and control condition (357). | Human | 61.4% Intervention group, 41.6% control | 16.1 | Sweden  (High income) | HPV | No | Single | |
| Hopfer (2009) | 400 | Treatment conditions; peer message (n 100), provider message (n 50), peer and provider message (100). Control conditions; video control (n 50), website control (n 50) and no message control (50). | Digital; Television | 100% | 21 | US  (High income) | HPV | No | Composite | |
| Meharry (2012) | 133 | Pamphlet condition (n 48), pamphlet and benefit statement condition (n 37) and control condition (n 49). | Printed material | 100% | Largest group 18-24 years | US  (High income) | Flu | Yes | Single | |
| Mehta et al (2013) | 90 | Health Belief Model based experimental condition (n 45) and control condition (n 45). | Human | 0% | Age range 18-25 years | US  (High income) | HPV | No | Composite | |
| Nan et al (2015) | 174 | First-person narrative (n 31), Third person narrative (n 40), Hybrid with first person (n 31) , Hybrid with third-person (n 34) and statistic control condition (n 38). | Digital; Computer | 50.90% | 20.5 | US  (High income) | HPV | No | Composite | |
| Payaprom et al (2011) | 201 | Health Action Process Approach with action planning Intervention condition (n 99), usual practice condition (102) | Printed material | 66.70% | 56.2 | Thailand  (Upper middle income) | Flu | No | Composite | |
| Peters (1995) | 115 | Experimental pre and post-test (n 38), experimental post-test only (20), control pre and post-test (37) and control post-test only (20). | Human | 60% | Age range 65- 93 years | US  (High income) | Flu and pneumococcal | No | Composite | |
| Prati et al (2012) | 311 | Narrative communication (n 100), didactic communication (n 103) and no message control condition (n 108) | Digital; Computer | 37.60% | 69.7 | Italy  (High income) | Flu | No | Composite | |
| Vet et al (2011) | 168 | Risk communication condition (n 37), social norm condition (n 37), combined condition (n 46) and no communication condition (n 48). | Digital; Computer | 0% | 33.8 | Netherlands  (High income) | Hepatitis B | No | Composite | |
| Worasathit et al (2015) | 2693 | Education group and a control group | Digital; Television | 80% | 69.5 | Thailand  (Upper middle income) | Flu | No | Single | |
| Wray et al (2009) | 111 | Vaccine safety message treatment condition (n 49) and vaccine information sheet control condition (n 59). | Printed material | 83% | Age range 50- 85 years | US  (High income) | Flu | No | Composite | |
