# Supplementary material for: Do interventions containing risk messages increase risk appraisal and the subsequent vaccination intentions and uptake? – A systematic review and meta‐analysis
Source: Br J Health Psychol. 2018 Sep 17;23(4):1084–106. doi: 10.1111/bjhp.12340 (PMC6767484; doi:10.1111/bjhp.12340)
Supplement: Supplementary file 5 — Table S3. Effect sizes of included studies, and BCTs in included studies. [file BJHP-23-1084-s005.docx]

Supplemental material 4:

Effect sizes of included studies, and BCTs in included studies

|  | Risk appraisal | | | | Intention | | | | Behaviour | | | | | BCTs* | |
| --- | --- | --- | --- | --- | --- | --- | --- | --- | --- | --- | --- | --- | --- | --- | --- |
| Study | Standard difference in mean | Standard Error | CI 95% | P Value | Standard difference in mean | Standard Error | CI 95% | P Value | Standard difference in mean | Standard Error | | CI 95% | P Value |  |  |
| Bennett  (Not included in Meta-analysis) |  |  |  |  |  |  |  |  |  |  |  | |  | Information about Health Cons’ |  |
| Dabbs et al 1966 (Not included in Meta-analysis) |  |  |  |  |  |  |  |  |  |  |  | |  | Information about health cons’ Information about emotional con’s (when pain level is high), Salience of cons’ (high fear). |  |
| De wit et al 2008: Narrative, combined severity and susceptibility | -0.228 | 0.357 | (-0.929, 0.473) | .524 | 0.822 | 0.320 | (0.196, 1.449) | .010 |  |  |  | |  | *No BCTs other than in control group* |  |
| De wit et al 2008: Statistical, combined severity and susceptibility | -0.321 | 0.305 | (-0.918, 0.277) | .293 | -1.612 | 0.346 | (-2.290, -0.933) | <.001 |  |  |  | |  | *No BCTs other than in control group* |  |
| Frew 2014: Susceptibility | 0.928 | 0.293 | (0.353, 1.503) | .002 | 0.010 | 0.208 | (-0.397, 0.418) | .960 | . | . | . | | . | *No BCTs other than in control group* |  |
| Frew et al 2013: combined Severity/ Susceptibility | 0.330 | 0.346 | (-0.349, 1.008) | .341 | 1.107 | 0.343 | (0.434, 1.780) | .001 | . | . | . | | . | Salience of Cons’. |  |
| Gerend et al 2012:  Susceptibility | 0.343 | 0.091 | (0.164, 0.521) | <.001 | . | . | . | . | -0.033 | 0.211 | -(0.447, 0.381) | | .875 | Information about emotional Cons’, anticipated regret, Information about social and environmental Cons’. |  |
| Godinho et al 2016:  Combined Severity/ Susceptibility | 0.187 | 0.083 | (0.024, 0.349) | .024 | 0.205 | 0.085 | (-0.038, 0.371) | .016 | . | . | . | | . | *No BCTs other than in control group* |  |
| Grandahl et al 2015: Combined Severity/ Susceptibility | 0.139 | 0.307 | (-0.463, 0.740) | .652 | . | . | . | . | -0.045 | 0.118 | (-0.277, 0.187) | | .703 | Information about health Cons’, Credible source. |  |
| Hopfer 2009:  Peer and provider; Susceptibility | -0.038 | 0.203 | (-0.436, 0.360) | .852 | . | . | . | . | -0.519 | 0.217 | (-0.944, -0.094) | | .017 | Information about Health Cons’ and Credible Source. |  |
| Hopfer 2009 Peer; Susceptibility | -0.175 | 0.166 | (-0.499, 0.150) | .292 | . | . | . | . | -0.381 | 0.210 | (-0.792, 0.031) | | .070 | Information about Health Cons’ and Credible Source. |  |
| Hopfer 2009: Provider; Susceptibility | -0.044 | 0.165 | (-0.367, 0.279) | .790 | . | . | . | . | -1.200 | 0.366 | (-1.918, -0.482) | | .001 | Information about Health Cons’ and Credible Source. |  |
| Meharry 2012: Combined Pamphlet; Severity/ Susceptibility | 0.095 | 0.248 | (-0.392, 0.582) | .703 | -0.959 | 0.498 | (-1.935, 0.017) | .054 | . | . | . | | . | Information about Health Cons’. |  |
| Meharry 2012: Combined Pamphlet and benefit; statement Severity/ Susceptibility | 0.271 | 0.26 | (-0.239, 0.781) | .298 | -0.441 | 0.475 | (-1.372, 0.490) | .353 | . | . | . | | . | Information about Health Cons’. |  |
| Mehta et al 2013: Combined Severity/ Susceptibility | 0.715 | 0.217 | (0.288, 1.141) | .001 | 0.462 | 0.214 | (0.043, 0.880) | .031 | . | . | . | | . | Information about health Cons’, Prompts/ cues, Information about social and environmental Cons’, Instruction on how to perform the behaviour. |  |
| Nan et al 2015: First person; Susceptibility | -0.209 | 0.372 | (-0.937, 0.520) | .575 | -0.130 | 0.372 | (-0.859, 0.598) | .726 | . | . | . | | . | Information about Health Cons’. |  |
| Nan et al 2015: Hybrid first person; Susceptibility | 0.624 | 0.377 | (-0.115, 1.364) | .098 | -0.199 | 0.372 | (-0.928, 0.529) | .591 | . | . | . | | . | Information about Health Cons’. |  |
| Nan et al 2015: Hybrid third person; Susceptibility | 0.823 | 0.377 | (0.084, 1.563) | .029 | -0.105 | 0.367 | (-0.824, 0.615) | .776 | . | . | . | | . | Information about Health Cons’. |  |
| Nan et al 2015: Third person; Susceptibility | -0.232 | 0.362 | (-0.941, 0.476) | .520 | -0.012 | 0.361 | (-0.721, 0.696) | .973 | . | . | . | | . | Information about Health Cons’. |  |
| Payaprom et al 2011: Combined; Severity/ Susceptibility | 0.103 | 0.141 | (-0.174, 0.380) | .466 | 0.278 | 0.237 | (-0.187, 0.743) | .241 | . | . | . | | . | Action planning, Goal setting (behaviour) Information about other's approval. |  |
| Prati et al 2012: Didactic; Combined measure of risk perception** | -0.068 | 0.172 | (-0.406, 0.270) | .693 | . | . |  |  | . | . | . | | . | Credible source |  |
| Prati et al 2012: Narrative; Combined measure of risk perception | 0.349 | 0.172 | (0.012, 0.686) | .043 | 0.062 | 0.164 | (-0.260, 0.384) | .705 | . | . | . | | . | Credible source |  |
| Peters 1995: Flu post; Combined Severity/ Susceptibility | -0.754 | 0.328 | (-1.396, -0.112) | .021 | . | . |  | . | 0.510 | 0.383 | (-0.241, 1.260) | | .183 | Information about social and environmental Cons’. Information about health Cons’ |  |
| Peters 1995: Flu pre/post; Combined Severity/ Susceptibility | -0.355 | 0.233 | (-0.812, 0.101) | .127 | . | . |  | . | 0.287 | 0.270 | (-0.242, 0.817) | | .287 | Information about social and environmental Cons’. Information about health Cons’. |  |
| Peters 1995: Pneumonia post; Combined Severity/ Susceptibility | -0.795 | 0.329 | (-1.439, -0.151) | .016 | . | . |  | . | 1.723 | 0.828 | (0.100, 3.346) | | .037 | Information about social and environmental Cons’. Information about health Cons’. |  |
| Peters 1995: Pneumonia pre/post; Combined Severity/ Susceptibility | -0.700 | 0.239 | (-1.167, -0.232) | .003 | . | . |  | . | 2.151 | 0.588 | (1.000, 3.303) | | <.001 | Information about social and environmental Cons’. Information about health Cons’. |  |
| Vet et al 2011: Combined condition; Susceptibility | 0.555 | 0.294 | (-0.022, 1.132) | .060 | 0.532 | 0.294 | (-0.045, 1.108) | .071 | . | . | . | | . | Risk communication: Information about health Cons’. Social norm communication: Information about other's approval. |  |
| Vet et al 2011: Risk message; Susceptibility | 0.561 | 0.304 | (-0.035, 1.158) | .065 | 0.362 | 0.301 | (-0.228, 0.952) | .230 | . | . | . | | . | Information about health Cons’. |  |
| Vet et al 2011: Social norm message; Susceptibility | 0.878 | 0.311 | (0.268, 1.487) | .005 | 0.669 | 0.306 | (0.069, 1.269) | .029 | . | . | . | | . | Information about other's approval. |  |
| Worasathit et al 2015: Combined Severity/ Susceptibility | 0.720 | 0.090 | (0.545, 0.896) | <.001 | 0.121 | 0.101 | (-0.076, 0.319) | .229 | . | . | . | | . | Information about health Cons’. |  |
| Wray et al 2009: Combined Severity/ Susceptibility | 0.315 | 0.194 | (-0.066, 0.696) | .106 | 0.022 | 0.193 | (-0.357, 0.401) | .909 | . | . | . | | . | Information about health Cons’. |  |

* BCTs depicted here relate to BCTs unique to the experimental condition.

** Due to insufficient sample size when calculating Intention effect size, only one condition was included in the meta-analysis.
