# Supplementary material for: Do interventions containing risk messages increase risk appraisal and the subsequent vaccination intentions and uptake? – A systematic review and meta‐analysis
Source: Br J Health Psychol. 2018 Sep 17;23(4):1084–106. doi: 10.1111/bjhp.12340 (PMC6767484; doi:10.1111/bjhp.12340)
Supplement: Supplementary file 6 — Table S4. Forest plots of outcome variables. [file BJHP-23-1084-s006.docx]

Supplemental material 5:

Forest plots of outcome variables

Forest plot showing meta-analysis for risk appraisal


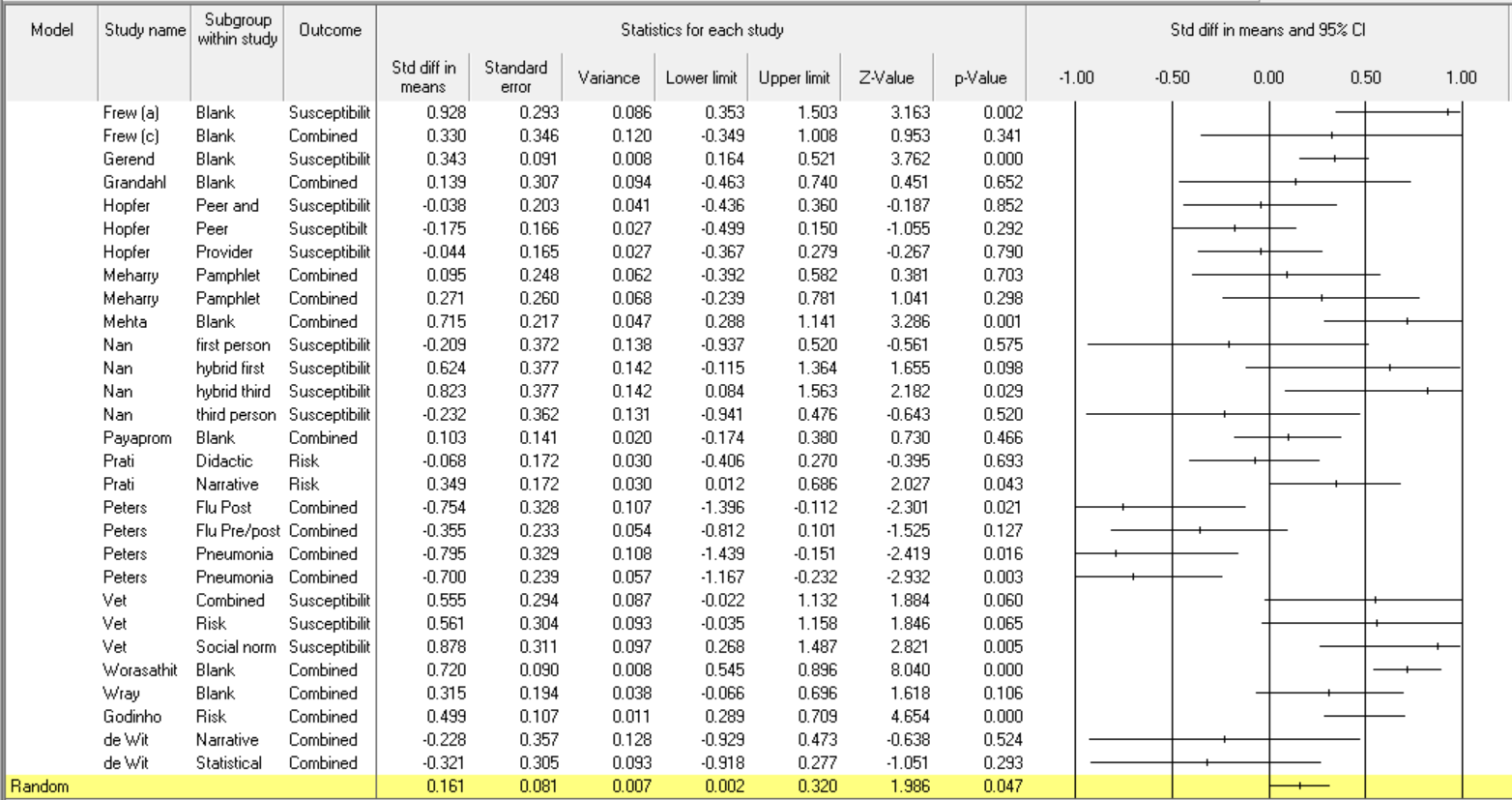


Favours Control Favours Intervention

Forest plot showing meta-analysis for intention


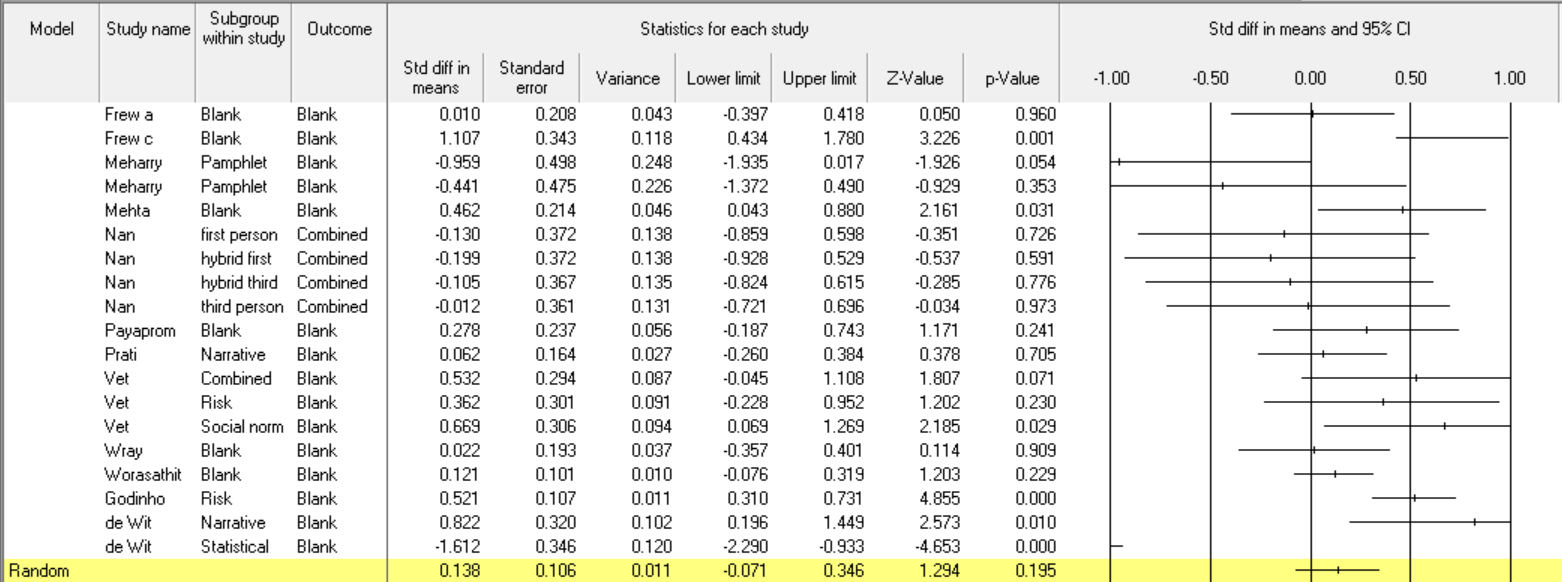


Favours Control Favours Intention

Forest plot showing meta-analysis for behaviour


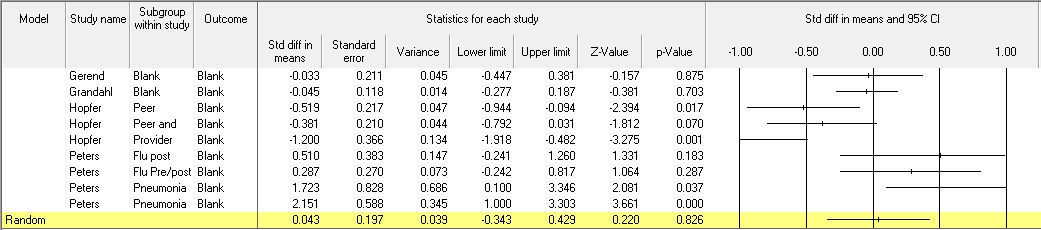


Favours Control Favours Intervention

Forest plot showing meta-analysis for the relationship between risk appraisal and intention


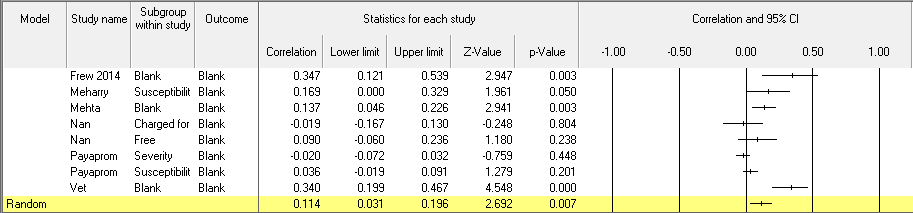


Favours Control Favours Intervention

Forest plot showing meta-analysis for risk appraisal- Susceptibility only


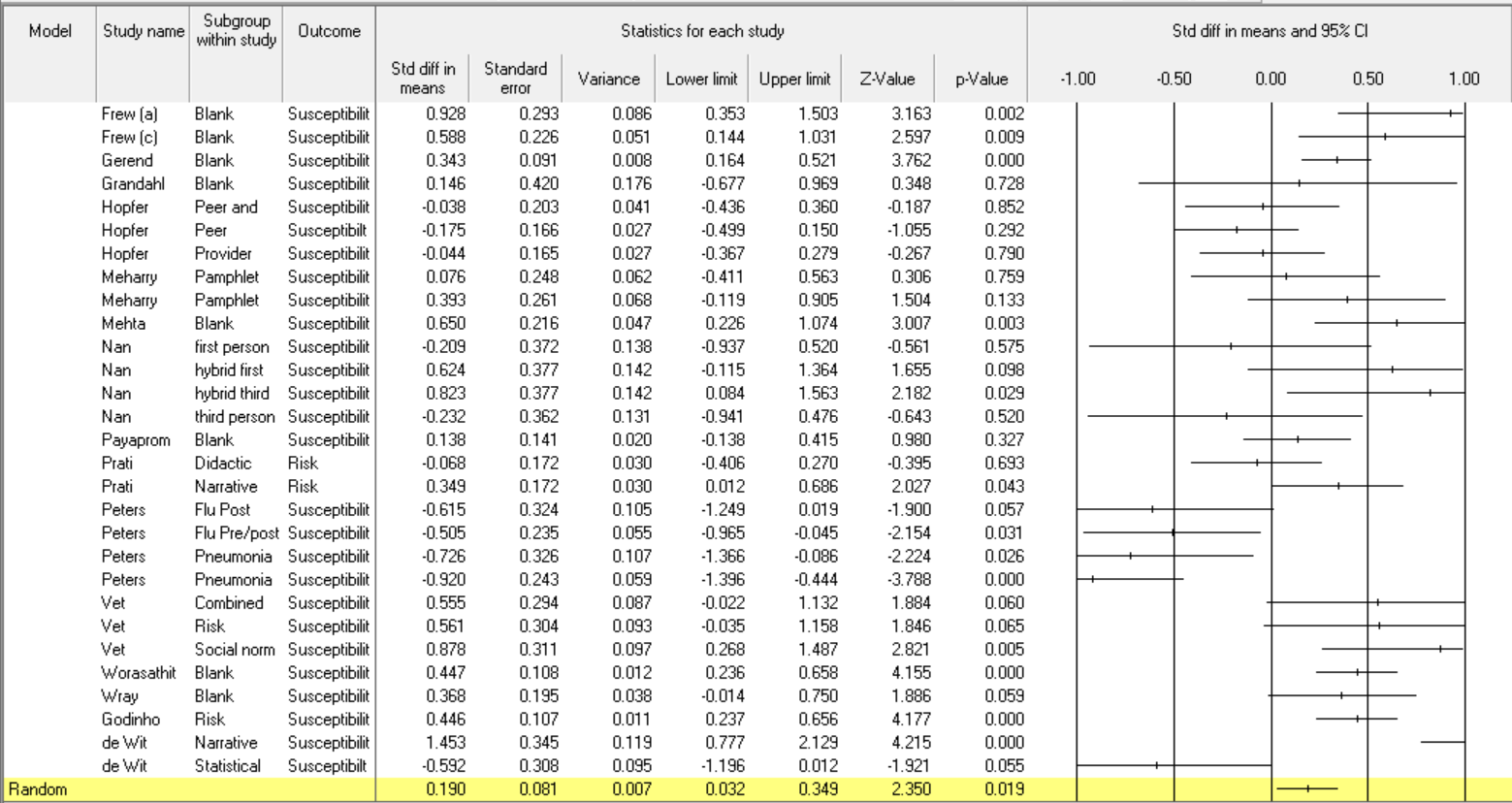


Favours Control Favours Intervention

Forest plot showing meta-analysis for risk appraisal- Severity only


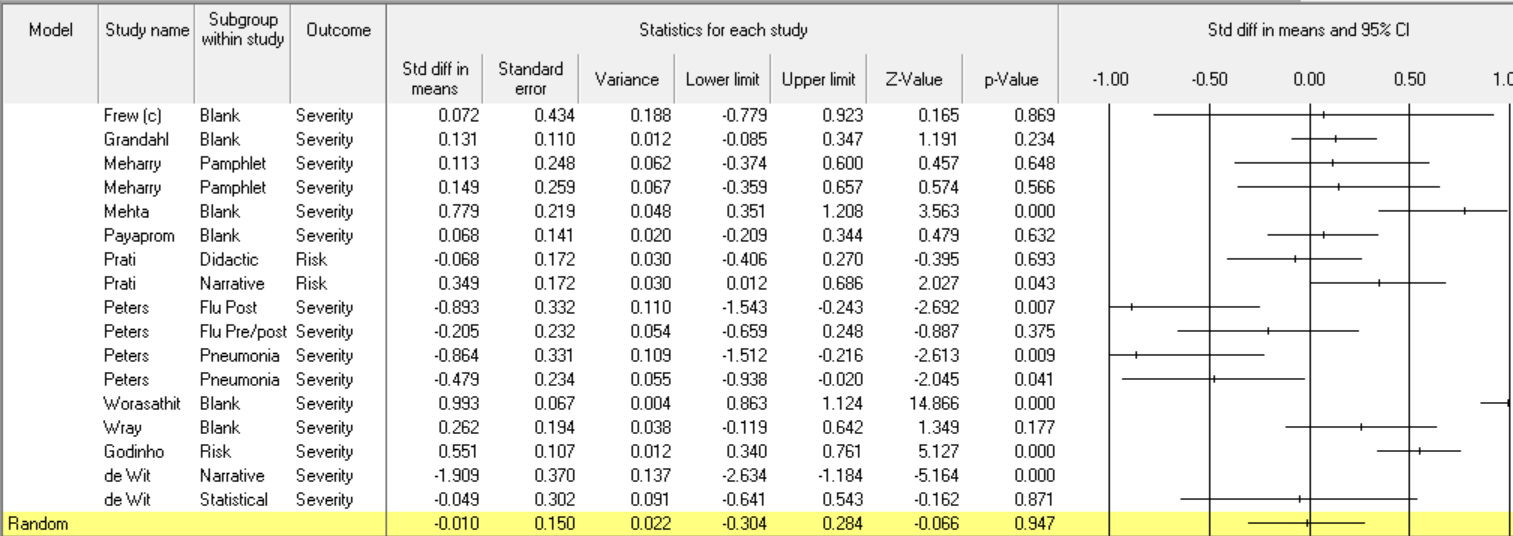


Favours Control Favours Intervention
