# Supplementary material for: Do interventions containing risk messages increase risk appraisal and the subsequent vaccination intentions and uptake? – A systematic review and meta‐analysis
Source: Br J Health Psychol. 2018 Sep 17;23(4):1084–106. doi: 10.1111/bjhp.12340 (PMC6767484; doi:10.1111/bjhp.12340)
Supplement: Supplementary file 7 — Table S5. Trim and fill adjusted values. [file BJHP-23-1084-s007.docx]

Supplemental material 7:

Trim and fill adjusted values

| Outcome Variable | Number of trimmed studies | Original value | Adjusted value |
| --- | --- | --- | --- |
| Risk | 0 | 0.161 | 0.161 |
| Intention | 0 | 0.138 | 0.138 |
| Behaviour | 2 | 0.043 | -0.193 |
